# Usability of a Technology-Based Bystander Bullying Intervention for Middle School Students in Rural, Low-Income Communities: Mixed Methods Study

**DOI:** 10.2196/32382

**Published:** 2021-10-26

**Authors:** Diana M Doumas, Aida Midgett, Valerie Myers, Mary Klein Buller

**Affiliations:** 1 Institute for the Study of Behavioral Health and Addiction Boise State Universtiy Boise, ID United States; 2 Department of Counselor Education Boise State University Boise, ID United States; 3 Klein Buendel Golden, CO United States

**Keywords:** technology-based bullying intervention, STAC-T, usability testing, middle school, rural, low-income, mobile phone

## Abstract

**Background:**

Students who are targets of bullying and who witness bullying are at high risk for negative mental health outcomes. Bystander training is essential to reduce bullying and the negative associated consequences for targets and bystanders. Resources necessary for program delivery, however, pose significant barriers for schools, particularly those in rural, low-income communities. Technology-based programs can reduce health disparities for students in these communities through cost-effective, easy-to-disseminate programming.

**Objective:**

The aim of this study is to conduct usability testing of a bystander bullying web app prototype, STAC-T (technology-based STAC, which stands for the 4 bystander strategies Stealing the Show, Turning it Over, Accompanying Others, and Coaching Compassion) as an initial step in the development of a full-scale STAC-T intervention. Objectives include assessing usability and acceptability of the STAC-T prototype, understanding school needs and barriers to program implementation, and assessing differences in usability between school personnel and students.

**Methods:**

A sample of 16 participants, including school personnel and students recruited from 3 middle schools in rural, low-income communities, completed usability testing followed by a qualitative interview. Descriptive statistics, 2-tailed independent sample *t* tests, and consensual qualitative research were used to assess usability and program satisfaction and to extract themes related to acceptability, feasibility, needs, barriers, and feedback for intervention development.

**Results:**

Usability testing indicated that the app was easy to use, acceptable, and feasible. Both school personnel (mean rating 89.6, SD 5.1) and students (mean rating 91.8, SD 7.0) rated the app well above the standard cutoff score for above-average usability (ie, 68), and both school personnel (mean rating 5.83, SD 0.41) and students (mean rating 6.10, SD 0.57) gave the app high user-friendliness ratings (0-7 scale, with 7 as high user-friendliness). The overall ratings also suggested that school personnel and students were satisfied with the program. Of the 6 school personnel who said they would recommend the program, 1 (17%), 4 (66%), and 1 (17%) rated the program as 3, 4, and 5 stars, respectively; 80% (8/10) of students said they would recommend the program; and 60% (6/10) and 40% (4/10) rated the program as 4 stars and 5 stars, respectively. Qualitative data revealed that school personnel and students found the STAC-T app to be useful, user-friendly, and relevant, while providing feedback related to the importance of digital learning activities that engage the user. Data from school personnel also indicated positive perceptions regarding program feasibility and probability of program adoption, with the most significant barrier being cost, suggesting the importance of considering the financial resources available to schools in rural, low-income communities when setting the price point for the full-scale STAC-T intervention.

**Conclusions:**

This study provides support for the full-scale development of the STAC-T app and provides key information for revision to enhance used engagement.

**Trial Registration:**

ClinicalTrials.gov NCT04681495; https://clinicaltrials.gov/ct2/show/NCT04681495

## Introduction

### Background

Bullying is a national public health issue in the United States, with 20.2% of students aged 12 to 18 years reporting being bullied at school and 15.3% reporting being cyberbullied in the previous year [1]. Both bullying and cyberbullying peak in middle school, with 28% of students reporting being a target of school bullying, and 33% reporting being cyberbullied [2]. Among middle school students, bullying victimization is associated with a variety of mental health problems, including somatic symptoms [3-7], anxiety [6-8], social anxiety [9-11], depression [5-9], suicidal ideation, and suicide attempts [8]. Similarly, being a target of cyberbullying is associated with internalizing symptoms and suicidal ideation [12-14]. Thus, the development of effective interventions for middle school students is important for reducing bullying and its negative consequences.

### Youth in Rural and Low-Income Communities

Youth in rural [15-17] and low-income [18-20] communities are particularly vulnerable to school bullying and cyberbullying. US national data indicate a higher prevalence of school bullying victimization among students in rural areas (23.8%) compared with students in urban areas (19.9%) [1]. Furthermore, students in rural areas are 3% to 5% more likely to report bullying their peers [17]. Students at the lowest income levels report the highest rates of bullying (21%-26.6%) compared with students at higher income levels (16.6%-19.8%) [1]. Students from low-income households also report the highest rates of physical bullying, bullying-related injury, and cyberbullying, yet they are the least likely to report bullying to an adult [1]. In addition, low-income students report the highest rates of consequences associated with bullying victimization, including negative effects on school work, relationships, feelings about oneself, and physical health [1]. Among middle school students attending schools in rural, low-income communities, bullying victimization is associated with poor school relationships, negative school experiences [19], and depression and anxiety [19,21]. These data reveal significant mental health disparities for youth in rural and low-income communities.

### Bullying Bystanders

Negative consequences are not only limited to targets of bullying but also extend to students who witness bullying [7,22] and cyberbullying [23-26] as bystanders. Students who witness school bullying are at increased risk for mental health problems, including somatic symptoms [7], sadness [27], helplessness [7,27], isolation, guilt [28], depression, anxiety [7,22], and suicidal ideation [7]. Similarly, witnessing cyberbullying is associated with anxiety, depression [23,25,26], and somatic symptoms [25], even when controlling for the effects of witnessing school bullying [23,25]. In addition, bystanders who intervene in bullying situations experience higher rates of anxiety and depressive symptoms than students who remain passive [29,30], possibly owing to using maladaptive behaviors to defend targets [30]. Research indicates 80% of students report witnessing bullying [7] and more than 50% witnessing cyberbullying [31]. Therefore, developing interventions to teach student bystanders how to appropriately intervene is important, as most students are bullying bystanders.

### School-Based Bullying Interventions

Results from a recent meta-analysis indicate that training student bystanders to intervene as *defenders* is an important component of comprehensive school-based bullying programs [32]. Although up to 80% of students report witnessing bullying [7], only 20% to 30% intervene [33]; for cyberbullying, as few as 10% may intervene [34]. Researchers have demonstrated that when bystanders are trained to act, it not only reduces school bullying but also leads to improved mental health in bystanders [35-39]. Bystander disapproval of cyberbullying acts can also effectively limit its prevalence [40]. Furthermore, self-efficacy for defending is positively related to intervening in both bullying [41] and cyberbullying [42]; however, few comprehensive, school-based programs incorporate bystander training.

In addition, there are several barriers to implementing school-based bullying prevention. Available interventions require substantial resources, including demands on teachers, limited access to training, lack of funding, and few school mental health professionals [43], reducing access and posing significant barriers to implementation. Rural, low-income communities face economic disparities, creating further obstacles to implementing these programs [44]. These barriers include a lower tax base for funding programs, training costs inflated by transportation needs related to bringing in expert trainers, frequent staff turnover with limited resources to reestablish expertise, school closures, staff overload and burnout, and lack of program advocates and local expertise in bullying prevention [16]. Therefore, brief programs that focus on bystander training and reduce barriers for implementation are needed to reduce bullying and its negative consequences among middle school students in rural and low-income communities.

### Technology-Based Interventions

National data in the United States indicate that the number 1 barrier to implementing educational technology is that school districts do not have the funding [45]. Technology-based interventions may be one way to increase access and reduce implementation challenges specific to rural and low-income communities [16]. Computer access and internet connectivity in middle school is high; 86% of students have computers in school [46], and only 6% of schools do not meet federal connectivity marks for broadband capacity [47]. Notably, low-income students have similar access to school computers compared with middle to high income peers [46]. Some rural areas have higher rates of poor internet connectivity; however, to assist in reducing this disparity, eligible rural and low-income schools receive discounts ranging from 20% to 90% for telecommunications services, including internet access and broadband services through the Telecommunications Act of 1996 [48]. Grants are also available through the US Department of Agriculture to build broadband infrastructure in rural areas [49], and more recently, the US Department of Education for discounted internet service for low-income households [50]. Thus, most students in rural, low-income communities have access to the infrastructure needed for technology-based programs.

### The STAC Intervention

STAC, which stands for the 4 bystander strategies Stealing the Show, Turning it Over, Accompanying Others, and Coaching Compassion, [51] is a brief, stand-alone bystander intervention developed to reduce barriers to program implementation for middle school students. STAC was originally designed as a 90-minute in-person intervention, including didactic and experiential role-play components, as well as 2 booster sessions. The didactic training includes education about bullying and cyberbullying, consequences of bullying, bystander roles, and a description of the 4 STAC strategies: (1) *stealing the show*—using humor or distraction to interrupt the bullying situation, removing the attention away from the target; (2) *turning it over*—informing an adult about the bullying and asking for help; (3) *accompanying others*—befriending or providing supporting the targeted student; and (4) *coaching compassion*—gently confronting the perpetrator to increase empathy for target. The experiential component includes role-plays in which students practice using the STAC strategies in hypothetical bullying situations. Students also participate in 2 biweekly 15-minute booster sessions to reinforce their skill acquisition. Research indicates that the STAC intervention is effective in reducing bullying [52,53] and negative mental health outcomes for bystanders [35-39]. Researchers have also adapted the STAC intervention to be culturally appropriate for middle school students in rural, low-income communities [54-56] with similar positive effects on bullying reduction [54,57] and improved mental health [54,58].

### Need for a Technology-Based STAC Intervention

Most bullying programs are comprehensive school-wide interventions that require significant resources, creating implementation barriers for schools. Although the STAC intervention reduces some of these barriers, in-person interventions pose implementation challenges, particularly for schools in rural, low-income communities. To increase access for these schools, we propose to translate the STAC intervention into a technology-based format. As a first step, we conducted a needs assessment with the goal of understanding needs and perceived program implementation challenges for schools in rural, low-income communities to provide information on how to best serve students in these schools [59]. Findings from interviews and focus groups with key school personnel (ie, administrators, teachers, and school counselors) from 3 middle schools in rural, low-income communities indicated a strong interest in a technology-based bullying intervention. Participants also described positive conditions for implementation, including support from the administration and technology readiness of their schools. Participants identified implementation challenges, such as time and financial resources. In addition, participants provided feedback related to translating the intervention into a technology-based format, including the importance of activities that require user input to increase user engagement. Overall, the findings supported the need for the proposed technology-based STAC (STAC-T) intervention and provided feedback on challenges that need to be addressed for successful adoption and sustained implementation in middle schools in rural, low-income communities.

### STAC-T Intervention

The STAC-T web-based app is intended to shift program delivery from an in-person intervention to a technology-delivered intervention, thereby increasing accessibility and eliminating implementation barriers. STAC-T will also allow large groups of students to be trained simultaneously, accessed from a computer, tablet, or smartphone. The STAC-T app is designed to be a modular program that can be customized to meet the needs of individual schools. The app will allow students to customize their experience by selecting avatars and bullying scenarios. Assessment and personalized feedback components are also infused through the program to individualize the user experience to promote behavior change [60,61]. The innovative, user-centered design of the STAC-T app will be inherently sensitive to the cultural needs of students and identify personally appropriate strategies. The STAC-T app addresses both bullying and negative mental health outcomes for targets and bystanders through an evidence-based approach that is adapted for a broader audience and uses technology to effectively implement bullying prevention.

The success of the STAC-T design can be evaluated through usability testing. Usability testing is an important step in the development of technology-based programs, providing information from end users on what works and gaps in how the program functions [62], as well as the acceptability and relevance of program content. Usability ultimately affects the likelihood that the program will be adopted [63] and thus is a critical component of technology-based intervention development [64]. Assessing usability with school personnel and students is also important, as school personnel make decisions about program adoption [65], and students, as end-users, need to understand and respond positively to the program to benefit from the content [62,66].

### Study Objectives

This study aims to evaluate the usability and acceptability of the STAC-T prototype to inform full-scale development of the STAC-T intervention app. To achieve this aim, we implemented usability testing with key stakeholders (ie, school personnel and students) at 3 middle schools in rural, low-income communities (N=16). The study had the following objectives: (1) to assess the usability and acceptability of the STAC-T prototype, (2) to understand school needs for and barriers to program implementation, and (3) to assess the differences in usability between school personnel and students. We used a mixed-methods design to assess the usability and acceptability of the app prototype, as well as ways to improve the app, likelihood of program use, and potential implementation barriers.

## Methods

### Participants

Participants were key school personnel (ie, administrators, teachers, and school counselors) and students were recruited from 3 middle schools in rural, low-income communities in the Northwest region of the United States. The schools were selected based on previous and ongoing research partnerships. The 3 schools were Title 1 schools, with 52.9% (339/641), 68.98% (725/1051), and 98.8% (663/671) of the student population at the 3 schools being below the poverty line. A total of 6 school personnel and 10 students were recruited. Among the school personnel, the age ranged from 28 to 59 years (mean 42.0 years, SD 10.8 years) and the majority were women (5/6, 83%) and White (6/6, 100%). For students, age ranged from 11 to 14 years (mean 12.2 years, SD 0.9 years), with 40% (4/10) in grade 6, 30% (3/10) in grade 7, and 30% (3/10) in grade 8, and the majority were girls (8/10, 80%) and White (7/10, 70%).

### Development of the STAC Prototype App

We used a multitheoretical framework to guide prototype production. We translated the STAC in-person intervention into a technology framework guided by Persuasive System Design, a theoretical guide for translating clinical aims to health-related technology frameworks [67-69]. Instructional objectives and design of STAC-T were guided by the existing STAC intervention for rural and low-income students, and input from an expert advisory board, school personnel, and students. The STAC-T prototype was developed using AGILE programming, a collaborative and incremental programming methodology [67-69]. The prototype was functional on all web browsers that support HTML5 and was built on a full stack web application using HTML or JavaScript as the main interface. React.js was used as the front-end framework. The system was accessible on desktop computers, iOS, Android tablets, and smartphones. Design ideas were created in written form, combined with scripts, flowcharts, and storyboards, before creating actual images and authoring elements. Programmers produced the STAC-T prototype, alpha- and beta tested it in-house for stability and code errors, tested it for usability, and revised it following an iterative, agile production process.

**Figure 1:**
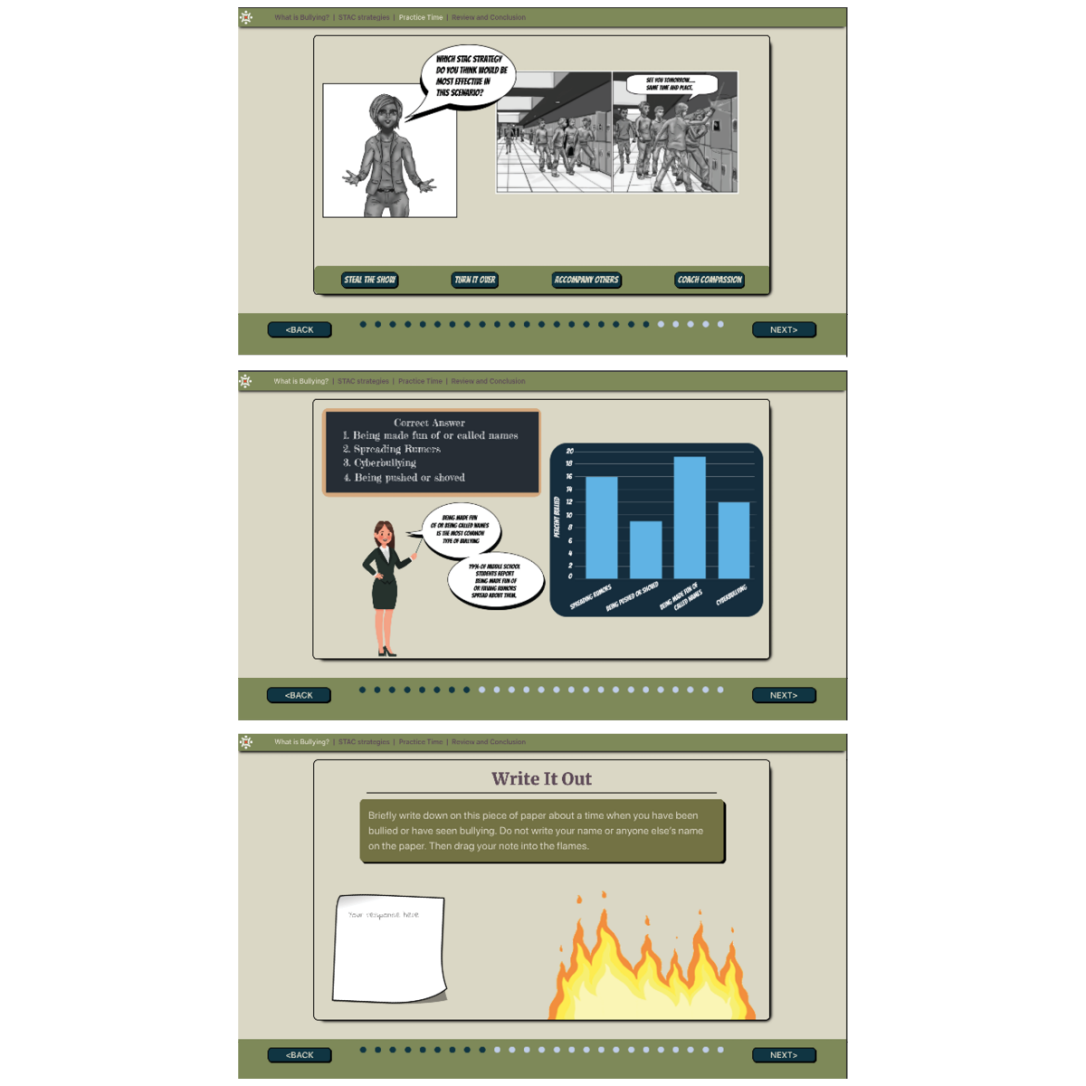
Samples from the STAC-T (technology-based Stealing the Show, Turning it Over, Accompanying Others, Coaching Compassion) prototype.

The initial prototype was created with clickable wireframes that showed the progression of content with still graphics (Figure 1). In addition to Persuasive System Design, User-centered Design [70,71], and the ADDIE (Analyze, Design, Develop, Implement, and Evaluate) model [72-74], we followed a user interface and instructional design approach to develop the STAC-T prototype. User input was solicited through iterative cycles, with adjustments made based on feedback [75], resulting in enhanced user experience and more reliable and effective results [76,77]. Design elements such as space (colors and visual space), components (characters and objects), and mechanics (actions) were determined for program features (ie, activities and games). Consistent with research on mobile health strategies for adolescents [78-80], the program development approach emphasized gaming as a teaching strategy, and interactive components were the centerpiece of the STAC-T intervention. STAC strategy practice required students to select between 2 avatars, view a bullying event, select actions to operationalize the STAC strategy, view the avatar enacting the selected action, and receive feedback on its effectiveness. To reward learning and bolster adherence, *badges* (visual reward icons eg, *Stealing the* *Show badge*) were included for intermittent awards to encourage user engagement.

The STAC prototype content comprised 3 overarching modules: (1) What is Bullying? Users were presented with background information on bullying, including bullying definitions (ie, physical, verbal, relationship, and cyberbullying), bullying facts and statistics, characteristics of students who bullied, and negative consequences of bullying; (2) What are Bystanders? Users were taught what a bystander is, and how bystanders affect bullying outcomes. This module explained the 4 bystander roles: (1) *Assistants*: those who intentionally help the bully; (2) *Reinforcers*: those who are not directly involved in hurting another student, but encourage the bully by standing around, laughing, or watching quietly; (3) *Outsiders*: those who do not take sides while witnessing bullying; and (4) *Defenders*: those who do something to stop the bullying situation or help the target in some way, and (3) STAC strategies: Users were introduced to 4 STAC strategies: (1) stealing the show, (2) turning it over, (3) accompanying others, and (4) coaching compassion. The module also included the STAC strategy practice using avatars selected by the user.

Iterative focus groups (3 rounds) with middle school students (N=21; 14 girls and 7 boys) attending schools in rural, low-income communities informed prototype development. Groups ascertained whether the app concept was easily understood and engaging and identified essential features for a successful prototype. The first round (n=10) was conducted with students who had been trained in the in-person STAC intervention. The second (n=6) and third (n=5) rounds were conducted with students who were naive to the STAC program. Group 1 provided feedback on the translation of the in-person STAC program content to a technology-based format for prototype 1. Group 1 reported that the content was realistic, expressed interest in a web translation, and made suggestions for modifications for web translation that were incorporated into prototype 1. For group 2, prototype 1 was very well received; participants thought the content was useful, important, and understandable and were enthusiastic about using the program. Group 2 participants suggested more interactivity (eg, short clips, videos, thought bubbles, graphs, and cartoons) to increase engagement. Group 3 evaluated prototype 2, reporting that they liked the program content and ease of navigation. Participants liked the sketches and discussed changes to characters (eg, wear hoodies instead of clothes with buttons) and surroundings (eg, school bus looked too clean) to more closely match the middle school environment. Input from the focus groups informed the development of the final prototype used in this study*.*

### Procedures

Participant recruitment and usability testing occurred during the spring of 2020. All research procedures were approved by the University Institutional Review Board and by the School District or Administration. The researchers provided the school counselor from each school with an email script describing the purpose and procedures of the study. School counselors were also provided with rubrics developed by the research team to identify key school personnel and students who demonstrated the following characteristics assessed by the rubric: school personnel: (1) caring for students, (2) desire to be a positive influence on school climate, (3) approachable to students, (4) caring about addressing the problem of bullying, and (5) leadership qualities; and students: (1) leadership, (2) maturity, (3) responsibility, (4) caring toward others, (5) influence, and (6) a desire to be a positive influence on peers. For each item, school personnel and students were assessed on a 3-point scale, which included the ratings of *yes* and *somewhat* to *no* for each item described above. School personnel and students who scored *yes* or *somewhat* on all inclusion criteria were eligible to participate. The school counselor used the rubric to identify and contact key school personnel and students and then used the script to invite them to participate in the study. Usability testing and interviews were conducted remotely. Researchers obtained informed consent for school personnel and parental consent and student assent for students and collected demographic information from participants immediately before usability testing.

Participants interacted with the STAC prototype app by selecting an avatar and environment to tailor content and talked aloud while completing the tasks and identifying problems and solutions attempted. Researchers and users were on videoconference and shared screens. Researchers could see what participants were doing, and they were able to communicate with each other in real time. The researchers observed the users as they worked through the tasks and asked questions to gather more data. Participants were asked to complete a brief usability survey followed by a semistructured interview protocol. All participants were asked to provide information about their perceptions of (1) program utility, (2) relevance and appropriateness of program content, and (3) ways they would improve the program. School personnel were also asked about (1) their thoughts on implementation feasibility, (2) likelihood of school program adoption, and (3) barriers to program use. All individual interviews lasted for 1 hour and were audio-recorded. School personnel received a web-based US $50 Amazon gift card as an incentive for participation in the usability testing and individual interview. There were no incentives for student participants.

### Measures

#### Demographics

Participants self-reported their age, ethnicity, race, and gender. Students also reported their grade level.

#### Usability

Usability was assessed using the System Usability Scale (SUS) [81]. The SUS is a widely used 10-item validated tool used to measure the usability and acceptability of technology-based programs. Responses were measured on a 5-point Likert scale ranging from 0 (*strongly disagree*) to 4 (*strongly agree*). Items were summed, and the total was multiplied by 2.5, creating an overall SUS score ranging from 0 to 100. An SUS score of ≥68 was considered above average [82].

#### User-friendliness

One item was used to assess the user-friendliness of the program. Participants were asked to rate the user-friendliness with the question: *Overall, I would rate the user-friendliness of this program as...* with a 7-point scale ranging from 0 (*worst imaginable*) to 7 (*best imaginable*).

#### Program Satisfaction

Two items were used to assess the program satisfaction. Participants were asked the question, “Would you tell your friends/colleagues to use the program?” with response choices *yes*, *no*, and *don’t know*. Participants were also asked how many stars they would give the program (1 star being the lowest and 5 stars being the highest).

#### Interview Questions

Following the usability testing sessions, participants were asked a series of open-ended questions about the utility and relevance of the app prototype, as well as ways to improve the app, likelihood of program use, and potential implementation barriers. School personnel and students were asked the following: (1) Please talk about your perception of how useful this program could be to helping to address the problem of bullying at school, (2) Please share your thoughts on whether you think the content of this program is relevant and appropriate for students at your school and your community, and (3) Can you talk about ways that you would improve the program? School personnel were asked the following: (4) What are your thoughts on how practical or workable you think it would be to use this program at your school? (5) What do you believe is the likelihood that your school would use this intervention? and (6) What, if anything, would keep you from using this program?

### Data Analyses

#### Quantitative

Quantitative data from the questionnaires were analyzed using the SPSS version 25.0. Before conducting statistical analyses, the data were examined for outliers and normality, and all variables were within the normal range for skewness and kurtosis. Descriptive statistics were used and presented separately for the school personnel and students. We examined the differences between school personnel and students using 2-tailed independent sample *t* tests for continuous variables and chi-square analyses for categorical variables. All analyses were considered significant at *P*<.05.

#### Qualitative

Qualitative data from open-ended questions were analyzed separately for the school personnel and students. A team member who participated in conducting the usability tests transcribed the data verbatim. We used thematic analysis [83,84] to identify, analyze, organize, describe, and report themes found within the qualitative data. NVivo was used to track quotes and organize themes. Two trained master’s students with previous experience in qualitative data analysis and a faculty member with expertise in qualitative methodologies analyzed the data. Before analyzing the data, the analysts discussed their assumptions and expectations regarding possible findings. Themes for each question were determined based on consensus. The process began with each team member individually developing initial themes for each question for school personnel and students. Next, the team met 2 times in person and conducted email communications over a 4-week period to arrive at a consensus on themes and frequency categories supported by participant quotations. During the first meeting, the lead analyst trained the team on thematic coding procedures, and each analyst coded the data separately. Next, the team met again for a second time to share each of their themes, followed by team members commenting and voicing agreement or disagreement. The analysts relied on participants’ quotes to resolve disagreements. Once the team reached a consensus, an external auditor reviewed the interview transcripts and themes. Overall, the auditor agreed with the team’s findings but provided an alternative way to organize one of the findings. The analysts used email correspondence to discuss and incorporate the auditor’s feedback and obtain a final consensus for themes. The external auditor agreed with the team’s final themes. Interview data were deidentified to ensure anonymity, and quotes were identified by participant type (ie, school personnel or students).

## Results

### Quantitative Analysis

#### Usability

The usability scores of the SUS are presented in Table 1. Overall, the scores for both school personnel and students suggested a very high level of usability, functionality, and acceptability. As presented in Table 1, there were no differences in any of the individual items or the SUS total score between school personnel and students, with both participant groups scoring the STAC-T app at a very high level of usability.

**Table 1:** Means and SDs for the System Usability Scale (SUS) by school personnel and students (N=16)^a^.

	School personnel (n=6), mean (SD)	Students (n=10), mean (SD)	*t* test (*df*)	*P* value
I think that I would like to use the program frequently	3.33 (0.82)	3.40 (0.52)	−0.20 (14)	.84
I found the program to be more complex than it needed to be	0.67 (0.52)	0.40 (0.70)	0.81 (14)	.43
I thought the program was easy to use	3.50 (0.55)	3.80 (0.42)	−1.24 (14)	.24
I think that I would need the support of a technical person to be able to use this program	0.17 (0.41)	0.20 (0.42)	−0.16 (14)	.88
I found the various functions in the program were well put together with each other	3.33 (0.52)	3.50 (0.53)	−0.62 (14)	.55
I thought there was too much inconsistency in this program	0.17 (0.41)	0.40 (0.52)	−0.94 (14)	.36
I imagine that most people would learn to use this program very quickly	3.50 (0.55)	3.60 (0.70)	−0.30 (14)	.77
I found the program very awkward to use	0.17 (0.41)	0.10 (0.32)	0.37 (14)	.72
I felt very sure that I could use the program correctly	3.33 (0.82)	3.60 (0.52)	−0.81 (14)	.43
I needed to learn a lot of things before I could get going with this program	0.00 (0.00)	0.10 (0.32)	−0.76 (14)	.46
SUS total score	89.58 (5.10)	91.75 (6.98)	−0.66 (14)	.52

^a^Responses were scored on a 5-point Likert scale ranging from 0 (strongly disagree) to 4 (strongly agree).

#### User-friendliness

School personnel and students rated the program high regarding user-friendliness. For school personnel, scores on user-friendliness ranged from 5 to 6 (mean 5.83, SD 0.41). For students, scores on user-friendliness ranged from 5 to 7 (mean 6.10, SD 0.57). There were no differences in scores between school personnel and students in terms of user-friendliness (*t*_14_=−1.00; *P*=.33).

#### Program Satisfaction

The program satisfaction ratings are listed in Table 2. Overall ratings suggested that school personnel and students were satisfied with the program. There were no differences in scores between school personnel and students on program recommendation (*χ*^2^_1_=1.3; *P*=.24, or star ratings, *χ*^2^_1_=2.3; *P*=.31).

**Table 2:** Program satisfaction by school personnel and students (N=16).

Variables		School personnel (n=6), n (%)	Students (n=10), n (%)
**Recommend** **program**			
	Yes	6 (100)	8 (80)
	No	0 (0)	0 (0)
	Unsure	0 (0)	2 (20)
**Star** **rating**			
	1 star	0 (0)	0 (0)
	2 stars	0 (0)	0 (0)
	3 stars	1 (17)	0 (0)
	4 stars	4 (66)	6 (60)
	5 stars	1 (17)	4 (40)

### Qualitative Analysis

#### Overview

Qualitative feedback for the STAC-T prototype supported the quantitative findings and was very positive overall, with participants sharing the perception that the STAC app is useful, relevant, and appropriate, as well as ways to improve the program. In addition, school personnel shared positive thoughts about program feasibility, a high likelihood of program adoption, and implementation barriers. The results are presented below, organized by the following themes: (1) utility and user-friendliness, (2) relevance, (3) program feedback, (4) feasibility, and (5) program adoption.

#### Utility and User-friendliness

Regarding participants’ perceptions on the program’s utility, school personnel and students indicated that STAC-T is useful and delivers helpful educational content to intervene in bullying. For example, 1 teacher stated, “this program is definitely one that can give strategies to students that they can apply and they also have a variety of strategies that they can choose from.”

A student shared:

I thought it was pretty good. It gave lots of information; gave different ways to deal with the problem or kind of bullying.

Furthermore, students emphasized that the program allowed them to learn about bullying. One student reflected, “I think it’d be really really good cause there’s some things I didn’t know and I learned about it.”

School personnel also indicated that the program is user-friendly and straightforward, and that developing the program on the web is a strength. A school counselor indicated:

I love that it’s gonna be digital because that’s right now what we do. We compete with the Minecraft’s [videogames] of the world.

#### Relevance

Participants were also asked to share their thoughts regarding the relevance and appropriateness of the program content for students at their school and their community. School personnel indicated that the program is relevant and can help increase students’ understanding of relationships. For example, a school counselor stated, “I definitively think that it’s relevant, just like we talked about, especially the online portion.”

Another school counselor indicated that the program helps “...kids to continue to understand the differences...” (between people). Students also indicated that the program was age-appropriate. One student said:

I think it is very appropriate and relevant. I think it’s a great idea, and it has great examples so kids can see [bullying] if they haven’t seen it before or see different situations it [bullying] could be in.

Another student shared:

Yeah I think it’s really good cause I’ve had some programs that use really big words, and so you can’t understand, or it explains every word like you’re a little child. Your program was perfect, like right at my level.

#### Program Feedback

When asked for feedback regarding how to improve the program, school personnel suggested that it is important to follow through while making the program interactive. One teacher stated:

I’d have to kind of see how all of the avatars work together, how the animation works together, how relevant that would work with keeping students’ attention. Because right now currently I’d have to read everything and know students aren’t going to read everything.

A school counselor said, “I definitely think having, like you already talked about, the piece of it being interactive, definitely needs to have that.”

Students suggested adding colors, including realistic pictures. One student stated:

I would definitely add color to the pictures, that’s one thing. I bet that would catch somebody’s eye. I know people who have more creative minds tend to pay attention to color more, cause I’m one of those people.

#### Feasibility

School personnel were also asked to speak about the feasibility of the program implementation. Participants provided positive responses indicating that the program was user-friendly and would align with existing programs that address social and emotional development. One school counselor indicated:

...it’s very user-friendly, even for me who had struggles getting going. It really gives you very clear, concise steps to be able to move forward and directions of what you need to do.

A teacher stated, “So, for me I can easily put it into the current curriculum or the current places in which we are but I think that would be up to individual teachers.”

#### Program Adoption

Finally, when asked about the likelihood of program adoption, the school personnel indicated that their school would be open to using this program. One teacher stated: “I know I would really enjoy it personally because I think it’s the strategies that are important for kids to understand and be able to use and apply.”

A school counselor indicated, “I think our school has been pretty open to learning new or picking up new tools that would enrich out student population.”

The school personnel indicated that resource constraints were the major barriers to adopting the program. A school counselor shared:

Money, money, and cost. But honestly, as far as implementation, it’s simple because it’s online. It really does come down to the time and how much does it cost.

## Discussion

### Principal Findings

This study aims to examine the usability of a technology-based bystander bullying intervention designed specifically for middle schools in rural, low-income communities. We were particularly interested in perspectives from both middle school personnel and students, as these participant groups represent key stakeholders who are in the position of making decisions regarding bullying programming and are the end users of the program. We aimed to test the usability of the STAC-T prototype; assess program utility, user-friendliness, and relevance; and gain an in-depth understanding of the needs and challenges related to program adoption and feedback regarding program content and delivery. Quantitative analysis of the survey data demonstrated a very positive response to the program, which was supported by qualitative data from individual interviews. Overall, the results indicate that participants perceived the STAC-T app to be useful, user-friendly, and appropriate for students at their schools and reported high levels of satisfaction with the program. Findings from this study indicate that the STAC-T app is relevant and feasible for implementation in middle schools in rural, low-income communities.

The findings of this study support the usability of the STAC-T app. For both school personnel (mean 89.58, SD 5.10) and students (mean 91.75, SD 6.98), scores on the SUS indicated a very high level of usability, well exceeding the standard cutoff score of 68 [82]. The user-friendliness of the program was also rated very high by both school personnel, with all participants rating the STAC app at ≥5 on a 0 to 7 point scale. We found no differences between school personnel and students in terms of SUS scores or user-friendliness ratings, suggesting that all participants found the program to be highly usable. Qualitative data supported these results, with both school personnel and students indicating that they perceived the program to be user-friendly, as well as age-appropriate and relevant for middle school students in rural, low-income communities. Furthermore, both school personnel and students reported high levels of satisfaction with the program, with most participants indicating that they would recommend the program to others. These findings are particularly important, as usability and acceptability are associated with both program adoption and implementation [63].

Regarding feasibility of implementation, school personnel indicated that they believed that their school would use the STAC-T app if the intervention was cost-effective. This finding is consistent with research indicating that administrators in rural middle schools would support a technology-based bullying program [59] and parallels research on bullying prevention in rural communities, identifying cost as a barrier to program implementation [16,59]. These findings also echo research suggesting the number 1 barrier to implementing educational technology is the lack of school district funding [45] and that financial resources and program effectiveness are necessary conditions for program implementation and sustainability of delivery of school-based programs [65].

Both school personnel and students discussed STAC-T program strengths and considerations for increasing user engagement. Qualitative findings suggested that participants perceived the STAC-T app to be useful and helpful in addressing the problem of bullying. They also indicated that the content of the program was appropriate for their schools, confirming the need for bullying programming that teaches students skills to use to intervene in bullying situations. This finding is consistent with previous research in which school personnel in rural, low-income communities indicated a need for bystander training that teaches students strategies to intervene on behalf of targets of bullying and includes having students actively practice these strategies across different scenarios [59]. Regarding user engagement, participants suggested adding more color and more realistic pictures, as well as increasing interactivity. This feedback is consistent with research suggesting that the motivating elements of technology-based interventions, including program content, length, and interactivity, are important in promoting behavior change [85].

### Limitations

This study supports the usability, relevance, and feasibility of the STAC-T prototype, providing valuable information for the development of a full-scale STAC-T app. However, certain limitations of this study must be noted. Participants were recruited from 3 schools in rural, low-income areas from 1 state in the Northwestern region of the United States. Although participants were recruited from 3 different counties to increase generalizability, school personnel and students from different regions of the country may have a different perspective. Furthermore, most participants in this study were female, further limiting the generalizability of the study. Although variability was low in the quantitative data and we did not identify divergent responses among participant interview responses, interpretation of the results for males should be made cautiously as females comprised approximately 81% (13/16) of the sample. Further formative research conducted during the development of the full-scale STAC app should include purposeful sampling to ensure appropriate representation of male participants. Furthermore, although most participants were White (13/16, 81%), this parallels the ethnic and racial composition of rural communities in the United States [86]. It is also possible that social desirability influenced participants as they were aware that the goal of the study was to translate the in-person STAC intervention into a technology-based format.

### Implications

This study has important implications for the development of a technology-based bullying intervention that addresses the needs and challenges specific to middle schools in rural, low-income communities. First, participants provided very high usability ratings for the STAC-T prototype, with qualitative data supporting the usability, utility, and relevance of the program. In addition, participants indicated that program implementation is feasible as long as the program is cost-effective. These findings support the development of the full-scale STAC-T app, while keeping the price point as an important consideration. Program cost considerations are particularly important in rural schools, as they face significant financial challenges due to a lower tax base for funding programs [16]. Furthermore, to enhance program implementation and sustainability, sufficient resources must be available [65]. Thus, the STAC-T app needs to be designed to be low in cost to be successfully adopted by schools in rural, low-income communities.

The findings from this study provide a strong scientific premise for moving forward with a full-scale production of the STAC-T app and have implications for program development. The full-scale STAC-T app will include 3 core modules, including strategy training, in which students select customized avatars as they move through role-plays in which they practice the STAC strategies. On the basis of participant feedback on the STAC-T prototype, all core modules will be built to include gaming and features requiring user input (eg, drag and drop, hover, click and reveal, and video). Real instances of game dynamics and mechanics will include several options, such as badges, leaderboards, levels, points, achievements, avatars, content unlocking, quests, social recognition, teams, and tokens. These items target the social cognitive theory components of modeling, outcome expectancies, self-efficacy, self-regulation, identification, and reciprocity. Consistent with research on mobile health strategies for adolescents [78-80], this design emphasizes gaming as a teaching strategy. The outcomes of each game will be used to ensure information uptake and demonstrate comprehension (eg, users must achieve a targeted score to receive a reward), thus providing feedback to participants early and often. To reinforce learning and bolster adherence, *badges* (ie, visual icons), which are highly effective gaming tools used to encourage user engagement and build game *loyalty* [87,88], will be awarded intermittently. These components will be the centerpiece of the program and are designed to increase engagement and learning.

### Conclusions

Bullying is a significant public health issue for middle schools in rural, low-income communities. Schools in these communities face multiple and competing demands on their time and have limited access to training, funding, and mental health professionals to implement bullying programming. The findings of this study demonstrate the usability, relevance, and feasibility of a brief, technology-based bystander bullying intervention. This study provides support for the development of the full-scale development of the STAC-T app and provides information that can be used to enhance program usability while addressing the unique needs of schools in rural, low-income communities.

## Abbreviations

ADDIE: Analyze, Design, Develop, Implement, and Evaluate
STAC: Stealing the Show, Turning it Over, Accompanying Others, Coaching Compassion
STAC-T: technology-based STAC
SUS: System Usability Scale
